# Target and Non-Target Processing during Oddball and Cyberball: A Comparative Event-Related Potential Study

**DOI:** 10.1371/journal.pone.0153941

**Published:** 2016-04-21

**Authors:** Sarah Weschke, Michael Niedeggen

**Affiliations:** Department of Education and Psychology, Freie Universität Berlin, Berlin, Germany; Central Institute of Mental Health, GERMANY

## Abstract

The phenomenon of social exclusion can be investigated by using a virtual ball-tossing game called Cyberball. In neuroimaging studies, structures have been identified which are activated during social exclusion. But to date the underlying mechanisms are not fully disclosed. In previous electrophysiological studies it was shown that the P3 complex is sensitive to exclusion manipulations in the Cyberball paradigm and that there is a correlation between P3 amplitude and self-reported social pain. Since this posterior event-related potential (ERP) was widely investigated using the oddball paradigm, we directly compared the ERP effects elicited by the target (Cyberball: “ball possession”) and non-target (Cyberball: “ball possession of a co-player) events in both paradigms. Analyses mainly focused on the effect of altered stimulus probabilities of the target and non-target events between two consecutive blocks of the tasks. In the first block, the probability of the target and non-target event was 33% (Cyberball: inclusion), in the second block target probability was reduced to 17%, and accordingly, non-target probability was increased to 66% (Cyberball: exclusion). Our results indicate that ERP amplitude differences between inclusion and exclusion are comparable to ERP amplitude effects in a visual oddball task. We therefore suggest that ERP effects–especially in the P3 range–in the Oddball and Cyberball paradigm rely on similar mechanisms, namely the probability of target and non-target events. Since the simulation of social exclusion (Cyberball) did not trigger a unique ERP response, the idea of an exclusion-specific neural alarm system is not supported. The limitations of an ERP-based approach will be discussed.

## Introduction

Social exclusion is a topic which got particular attention in recent years in both social psychology and neuroscience. The seminal model of Williams and colleagues (e.g. [1, 2]) states that four fundamental social needs–belonging, self-esteem, meaningful existence, and control–are immediately threatened by social exclusion. These researchers have developed the now widely used Cyberball paradigm, a virtual ball-tossing game in which participants are included or excluded by presumed, but actually computer-generated “co-players” [3, 4]. This paradigm is particularly able to induce need threat [5], which is retrospectively measured by the Need Threat Questionnaire (NTQ, [3]), and it was frequently used in both behavioral and neuroimaging studies.

The first fMRI studies using the Cyberball paradigm [6, 7] found activation in the dorsal anterior cingulate cortex (dACC) and the anterior insula during social exclusion. These areas are also involved in the processing of physical pain [8]. The “social/physical pain overlap theory” was supported by behavioral [9] and pharmacological studies (see [10] for a review). Although the involvement of structures within the ACC in the processing of social exclusion was recently confirmed by a meta-analysis [11], critical voices stated that it can neither be concluded that social pain is experienced like physical pain nor that the overlapping activation pattern represents a pain-specific network. It was suggested that these areas are part of a “saliency network” or that they represent the processing of expectancy violations [12, 13]. Another meta-analysis showed that structures reliably activated during exclusion signaled social uncertainty, rumination, distress, and craving, but not social pain *per se* [14]. An alternative explanation for overlapping activation during physical and social pain was given by a recent study: although there are common activation patterns which were found by using gross analysis procedures, multivariate pattern analysis revealed subregions within these structures which were independently activated during physical pain and social rejection. Since these subregions have distinct functional connectivity patterns with other brain regions, it was concluded that pain and rejection distress are distinct types of affect [15].

Beside neuroimaging studies there are also electrophysiological studies which took advantage of the Cyberball paradigm (e.g. [16–20]). If the ball was passed from one co-player to another, these events triggered a typical sequence of event-related potentials: a negative peak at approximately 200 ms (N2) followed by a more sustained positivity peaking at about 350 ms (P3). Within the conceptual framework defined by Williams and colleagues, the components were associated with the activation of the neural alarm system [16, 17, 21]. Moreover, some results revealed a correlation between P3 amplitudes and perceived ostracism intensity or social pain [17, 18, 22]. But–corresponding to the fMRI research–the functional assignment of ERP components to social exclusion and the neural alarm system is questionable.

We suggest that it is more likely that the effects in the P3 range are mediated by the probability of ball possession or–more precisely–by the expectancy for ball possession [23]. In ERP literature, the processing of expectancy and stimulus probabilities is usually examined by using an oddball paradigm [24, 25]. Here, the frequency of a task-relevant *target* event is varied systematically. Following this approach, we used a partial exclusion condition in our previous studies allowing a separate analysis of ERP responses to the event “self” (= ball possession of the participant) during inclusion and exclusion. In line with an early Cyberball study [3], partial exclusion was found to be sufficient to trigger need threat and negative feelings [18]. Transferring the terms of the Cyberball to the oddball paradigm, the event “self” served as target stimulus because it requires a reaction from the participant, whereas the event “other” (= ball possession of a co-player) served as standard or *non-target* stimulus. In line with the established ERP signatures identified in the oddball paradigm [26], the parietal P3 (P300 or P3b) amplitudes were larger for target stimuli (event “self”) compared to non-targets (event “other”) [18, 22]. For both the events, “self” and “other”, the amplitude of the P3 complex (with a central P3a and a parietal P3b component) was affected by stimulus probability or expectancy for target or non-target appearance [18, 19, 23]. Similar ERP components were found to be modulated in independent Cyberball studies where analysis was focused on the events “other”, but these effects were not interpreted within the framework of the oddball paradigm [16, 17].

The striking similarity of ERP signatures triggered in the Cyber- and the oddball paradigm leads itself to the question whether a common process is triggered. Our aim was to further prove our interpretation of the ERP effects of exclusion in the Cyberball paradigm. Therefore we directly compared these two paradigms in the current study: we designed a visual oddball task which was physically identical to our ERP-compatible Cyberball paradigm (see [19], Presence group), but the social context was eliminated as participants assumed to take part in a simple reaction experiment. In a standard Cyberball condition, participants were told that they would play a ball-tossing game with two other co-players connected via internet. Data of this condition have already been presented in [19] (Internet group). Here, they serve as a control for the effects in the Oddball group. Participants of both groups ran through two consecutive blocks, with the second block defined by a decrease in target probability.

We suppose that the social cover story provided in the Cyberball design will specifically affect the retrospective evaluation in a questionnaire, but not the “online” processing of subjective expectancies. We propose that a feeling of ostracism will not emerge when the social context of the task is removed (group “Oddball”). Only in the Cyberball task a reduction of target probability (“ball possession”) is expected to induce social need threat and to increase negative mood.

As we have shown in our previous studies [18, 19], the event “self” in Cyberball evokes two distinct ERP components. We hypothesize that the same components will be identified if target events are presented in the Oddball paradigm.

The first component was a parietal N2 which was interpreted as a correlate of task relevance [27]: it was more pronounced after the event “self” which demands a motor reaction from the participant compared to the event “other”, but the amplitude did not differ between inclusion and exclusion [18]. Therefore this component should also not be influenced by the probability of the target stimulus in the Oddball task.

The second component was a P3 complex, consisting of a central P3a and a parietal P3b. Earlier results showed that these components are sensitive to ostracism manipulation in the Cyberball paradigm [17–19]. Although a correlation between P3a and negative mood, and P3b and ostracism intensity was found in one study [18], we have not been able to replicate these results [23]. We therefore propose that the ERP effects in the P3 range in the Cyberball and a visual oddball task rely on the same mechanism, namely the expectancy for target appearance (or ball reception), and expect similar effects of target probability on the P3 complex.

Previous ERP Cyberball studies realized a continuous exclusion of the participant, and therefore focused on the analysis of “other” events [16, 17, 20–22]. In order to apply our approach to these previous results, we extended our ERP analyses to these “non-target” events. Oddball studies show that the P3 complex elicited by non-targets has–possibly due to inhibition processes–a more anterior topography as compared to this component for target events [28], and that the amplitude is also modulated by stimulus probabilities [26]. We therefore expect greater amplitudes of the P3 complex elicited by non-targets during inclusion as compared to exclusion as a function of non-target probabilities. Explorative analyses will indicate if the reduction of the P3 complex from inclusion to exclusion is less pronounced in one of the two paradigms.

By directly comparing the ERPs elicited by target and non-target events in the Cyberball and Oddball task, we also have the opportunity to check if there are any components or experimental effects which are specifically evoked in one of the two paradigms.

## Materials and Methods

### Participants

Twenty-one healthy subjects took part in the Oddball task. Due to a high number of artifacts in the EEG or missing questionnaire data, six participants had to be excluded, leaving fifteen (10 female) for analyses. Their mean age was 21.7 years (*SD* = 2.9 years). In the Cyberball task, eighteen subjects took part. Three of them had to be excluded, leaving fifteen (7 female) for analyses. Their mean age was 24.7 years (*SD* = 6.8 years). Age did not differ significantly between groups, *F*(1,28) = 2.47, *p* = .128. The procedure was approved by the ethics committee of Freie Universität Berlin (Ethikkommission der Freien Universität Berlin). Participants were recruited in the university environment and got credit points for their studies, if desired. They gave their written consent for participating according to the Declaration of Helsinki. Since a cover story was required to induce the experimental effect in the Cyberball task, participants were informed about the experimental technique and aiming of the study immediately after the experiment. After that they again gave their written consent for participation.

### Stimuli and procedure

E-Prime 2 (Psychology Software Tools, Inc.) was used to present standardized instructions, the visual Oddball or the Cyberball task, and to trigger EEG recordings. The participants in the Cyberball group were told that they took part in a study testing visual imagination capabilities. To keep up this cover story, they first completed a short questionnaire about visual imagination (Vividness of Visual Imagery Questionnaire, [29]). The participants in the Oddball group were informed that they were taking part in a simple reaction experiment.

**Fig 1:**
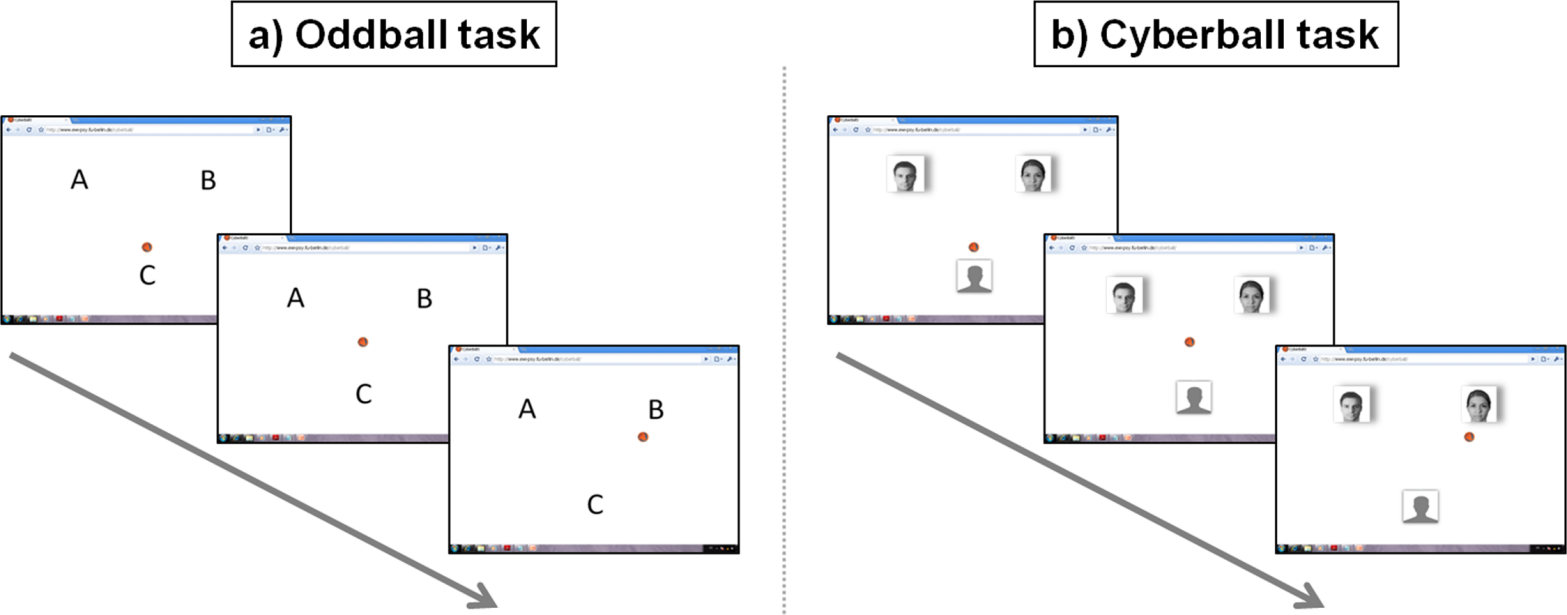
**Display of the visual Oddball (a) and the Cyberball (b) task.** The events “circle at position ‘C’” (Oddball) or “ball possession of the participant” (Cyberball) were defined as target events, since they required the participant to press a button to move the circle/ball to the position “A” or “B” or to the left or right co-player. Before the circle/ball appeared at the selected position, it remained at the center of the screen for 500 ms. At the positions “A” or “B” or at one of the co-players’ position (non-target events) the circle/ball remained for 400 to 1400 ms, and the participant was not required to react. Note that the pictures of the presumed co-players (b) were no real photos, but morphs of different persons.

The setup for the Cyberball group (see Fig 1A) followed an established EEG-compatible Cyberball design (see also: [19], Cyberball Internet group). Participants were told that they would play with two other players connected via internet. Photos of the presumed co-players were depicted on the screen. For the Oddball group, these photos were replaced by single letters at the corresponding positions (see Fig 1B). A physically identical display had also been used in our previous study ([19]: Cyberball Presence group).

After instructions and a short training introduction, participants of both groups went through two experimental blocks. There was a short break between the blocks. Each block consisted of 200 trials and lasted about seven minutes. One trial was defined as the appearance of a red circle that symbolized a ball in the Cyberball task at one of the designated positions (target or non-target, see Fig 1). The circle appeared at the center of the screen for 500 ms between target and non-target events. At a non-target position (position “A” or “B” or “ball possession of a co-player”), the circle remained for 400–1400 ms before it moved back to the center and then to the other non-target or to the target position. The target event was defined as the appearance of the circle at position “C” (Oddball) or at the position assigned to the participant (Cyberball). The onset of this stimulus required the subject to move the circle to another position by button press (“g” to move the circle to position “A” or to the left co-player, and “h” to move the circle to “B” or to the right co-player) on a commercially available keyboard. No speeded response was required.

In the first block, the probability of the target event was 33 percent (“inclusion”), in the second block it was reduced to 17 percent (“exclusion”). This parallels our previous work [18, 19]. Non-target events were defined by the (unpredictable) appearance of the non-targets at positions “A” or “B” or by the (unpredictable) ball reception of one of the co-players, i.e. trials in which the ball/circle was moved by the participants were not analyzed. Consequently, the probability of (unpredictable) non-target events was 33% in the first and 66% in the second block of the tasks.

After the second block, two Need Threat Questionnaires were handed out. The subjects were told to retrospectively fill out the questionnaires, the first one regarding the first block, and the second one regarding the second block. A German version of the NTQ was used (translation by [30]). Some items (e.g. “I felt like an outsider.” “I felt rejected.”) of this questionnaire are not appropriate for a non-social oddball task, and hence the validity is disputable. However, we decided to also apply it to the Oddball group to control for possible effects. There is evidence that need threat is evoked even in a situation where the participants knew that their co-players were computer-generated [31]. The participants were also asked to estimate the probability of the target stimulus (Cyberball: “ball possession”; Oddball: “circle at position ‘C’”) in the first and second block, respectively.

### EEG recording and analysis

EEG data were recorded from three active electrode positions (Fz, Cz, Pz) using Ag/AgCl skin electrodes which were fixed on the scalp with EC2 Electrode Cream (Grass Technologies). Active electrodes were referenced to linked earlobes with AFz serving as ground. Impedances were kept below 10 kΩ. Vertical and horizontal electrooculogram (vEOG, hEOG < 20 kΩ) was recorded to control for ocular artifacts. Biosignals were recorded with EEG BioAmplifiers and Psylab recording software (Contact Precision Instruments, London) and analyzed with BrainVision Analyzer 2 (Brain Products GmbH, Germany). EOG data were low-pass (30 Hz) and notch filtered (50 Hz) and EEG data were band-pass (0.3 to 30 Hz) and notch filtered (50 Hz) offline. EEG segments were created (-100 to 800 ms after target or non-target onset, separately for the first and the second block) and baseline-corrected (-50 to 50 ms after stimulus onset). Subsequently, a rigorous artifact rejection was performed. In a first step, all segments containing activity exceeding +/- 40 μV were automatically eliminated to control for eye blinks. In a second step, a manual artifact rejection was performed to exclude segments containing muscular or other artifacts, eye movements, slow drifts in the baseline, or high alpha activity. The number of kept segments (Cyberball: *M* = 55%, Oddball: *M* = 59%) did not differ between tasks, *F*(1,28) = 0.42, *p* = .52. Analyses focused on segments defined by the (unpredictable) appearance of the target or non-target. In almost all cases the number of kept segments after artifact rejection was lowest for target events in the second block. The number of EEG segments of the other conditions (targets in block 1, non-targets in block 1 and 2) was randomly chosen to adjust it to the number of segments obtained in the reference condition (i.e. the condition with the lowest number of kept segments, see above). Averages and grand averages were calculated separately for the two tasks, two blocks, two event types, and three electrode positions.

**Fig 2:**
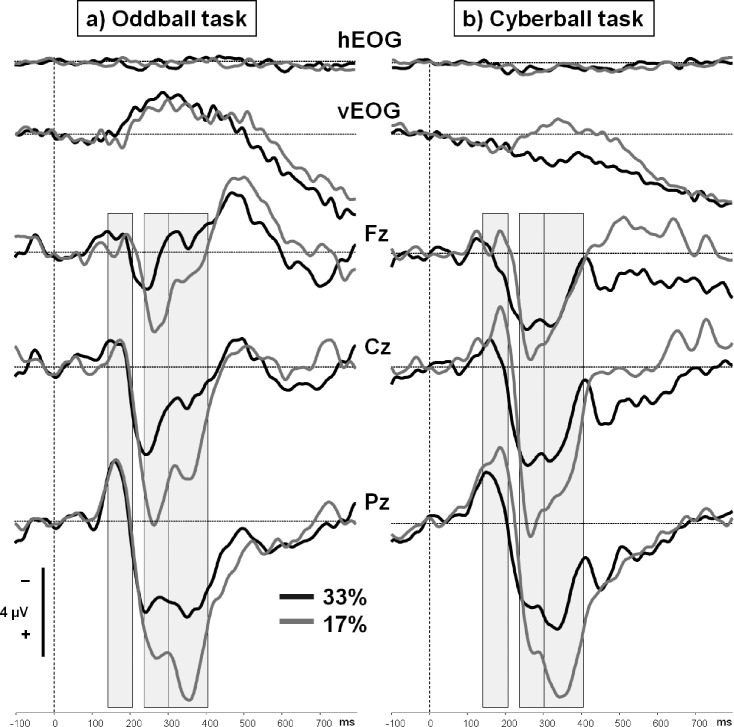
Grand-averaged ERPs for target events. Grand-averaged ERPs and horizontal and vertical EOGs for the target events (a: Oddball task, circle at position “C”; b: Cyberball task, ball possession of the participant) in the first (33% target probability; black lines) and the second (17% target probability; grey lines) block of the tasks. ERPs were recorded from the electrode positions Fz, Cz, and Pz. A parietal N2 (130–210 ms) was followed by a P3 complex with a centrally located P3a (240–300 ms) and a P3b (300–410 ms) with a parietal maximum. The target event was presented at 0 ms.

Grand-averaged ERPs elicited by target events revealed three distinctive components in both groups (see Fig 2): an initial posterior negativity (130 to 210 ms, N2), followed by a P3 complex with a central positivity at 240–300 ms (P3a), and a subsequent parietal positivity (300 to 410 ms, P3b). Sequence and time ranges of ERP components elicited in the Oddball group replicated our earlier Cyberball results [18, 19]. Visual inspection of the ERPs elicited by the target stimuli suggest a fronto-central enhancement of a late negativity (> 400 ms after target appearance) in the second block, but this effect was not found to be significantly modulated by the factor “task”. Therefore the late negativity will not be considered in detail in the result section. Since the grand averages also suggest a latency shift of the N2 component from the first to the second block in the Cyberball group, an analysis of peak latencies and amplitudes was conducted for this component. However, analyses did neither reveal an effect of block and/or task on peak amplitudes nor on peak latencies. Therefore we decided to restrict the report to the mean amplitudes of components in the predefined times ranges.

**Fig 3:**
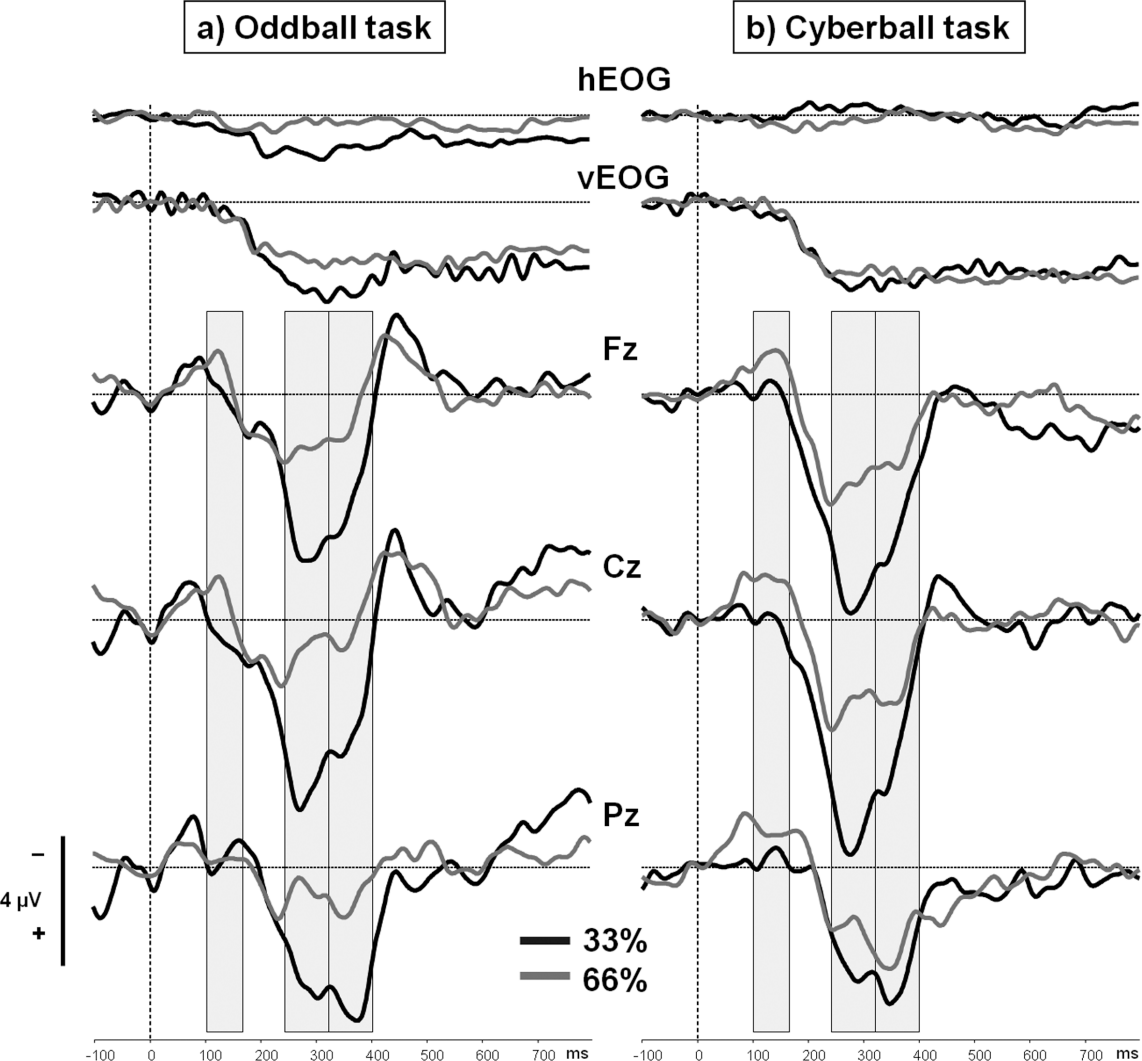
Grand-averaged ERPs for non-target events. Grand-averaged ERPs and horizontal and vertical EOGs for the non-target events (a: Oddball task, circle at position “A” or “B”; b: Cyberball task, ball possession of the co-players) in the first (33% non-target probability; black lines) and the second (66% non-target probability; grey lines) block of the tasks. ERPs were recorded from the electrode positions Fz, Cz, and Pz. An early N2 component (100–170 ms) was followed by a fronto-central P3 complex consisting of a P3a (240–320 ms) and a P3b (320–400 ms) component. The non-target event was presented at 0 ms.

Grand-averaged ERPs elicited by non-target events revealed an early N2 component (100–170 ms), followed by a fronto-central P3a (240–320 ms) and a P3b (320–400 ms) component (see Fig 3).

Both ERPs for target and non-target events did not reveal any components or effects which were specifically elicited in only one of the two paradigms.

### Data analysis

For each participant, mean amplitudes of the components of interest (see above) were exported and read into SPSS (version 21, IBM). Furthermore, questionnaire data were entered in SPSS, and NTQ scales “belonging”, “self-esteem”, “meaningful existence”, and “control”, and an additional scale included in the NTQ measuring negative mood were computed (all items were rated on a 1 to 5 Likert scale, with need scales having a potential range between 1 and 5 and negative mood between 4 and 20). Although a speeded response was not required in both tasks, reaction times were recorded and analyzed (participants’ throwing of the ball/moving of the circle to another position). To control for outliers, medians for each task and block were calculated for all participants. Data were analyzed running repeated measures ANOVA including the within-subjects factor “block” (target/non-target probability 33% (block 1) vs. target probability 17%/non-target probability 66% (block 2)) and–for ERP data–including the within-subjects factor “electrode position” (Fz vs. Cz vs. Pz) and the between-subjects factor “task” (Cyberball vs. Oddball). Multivariate tests will be reported. In addition, correlations between the experimental effects (Δ block 1, block 2) on questionnaire and ERP data were computed for both experimental groups (see S1 Table for *r* and *p* values). Only isolated and unsystematic correlations were found.

## Results

### Questionnaire and behavioral data

Participants noticed the difference of target occurrence between experimental blocks (see Table 1 for descriptive data), which was confirmed by a main effect of block, *F*(1,28) = 60.59, *p* < .001, η_p_^2^ = .68. However, this effect was significantly modulated by the factor “task”, *F*(1,28) = 5.47, *p* = .027, η_p_^2^ = .16, indicating that it was less pronounced in the Oddball group, *F*(1,14) = 20.29, *p* < .001, η_p_^2^ = .59, as compared to the Cyberball group, *F*(1,14) = 40.36, *p* < .001, η_p_^2^ = .74.

**Table 1:** Questionnaire and behavioral data of the Oddball and Cyberball group for the two experimental blocks.

	Oddball		Cyberball	
	Block 1	Block 2	Block 1	Block 2
**Estimated target occurrence (%)**	28.2 (11.3)	18.7 (5.6)	30.5 (10.4)	12.9 (5.5)
**Belonging**	4.11 (0.70)	3.98 (1.00)	3.80 (0.58)	2.53 (0.92)
**Self-esteem**	3.36 (0.54)	3.18 (0.60)	3.60 (0.61)	3.18 (0.50)
**Meaningful existence**	4.38 (0.82)	4.36 (0.85)	4.27 (0.83)	3.60 (1.09)
**Control**	2.36 (0.90)	2.20 (0.71)	2.31 (0.70)	1.67 (0.74)
**Negative mood**	9.40 (2.66)	9.80 (2.42)	8.53 (2.53)	12.60 (2.75)
**Reaction times (ms)**	563 (160)	524 (114)	672 (388)	755 (443)

Mean values and standard deviations (in brackets) are presented.

Descriptive data of the NTQ for the Oddball and Cyberball group are presented in Table 1. As we had already reported in our previous paper [19], there was a significant decrease in need satisfaction for belonging, *F*(1,14) = 19.64, *p* = .001, η_p_^2^ = .58, self-esteem, *F*(1,14) = 4.89, *p* = .04, η_p_^2^ = .26, and control, *F*(1,14) = 7.21, *p* = .02, η_p_^2^ = .34, and a nearly significant reduction for meaningful existence, *F*(1,14) = 4.12, *p* = .06, η_p_^2^ = .23, after exclusion in the Cyberball group. Negative mood significantly increased after the second block, *F*(1,14) = 21.99, *p* < .001, η_p_^2^ = .61. In the Oddball group, a significant difference between blocks was obtained in none of the scales (belonging: *F*(1,14) = 0.63, *p* = .44, η_p_^2^ = .04; self-esteem: *F*(1,14) = 4.35, *p* = .06; η_p_^2^ = .24, meaningful existence: *F*(1,14) = 0.02, *p* = .89, η_p_^2^ = .001; control: *F*(1,14) = 1.15, *p* = .30, η_p_^2^ = .08, negative mood: *F*(1,14) = 0.69, *p* = .42, η_p_^2^ = .05).

In the Oddball task, reaction times were slightly faster in the second block (block 1: *M* = 563 ms, block 2: *M* = 524 ms), but this was not statistically significant, *F*(1,14) = 4.08, *p* = .06. In the Cyberball task, reaction times were prolonged in the second block (block 1: *M* = 672 ms, block 2: *M* = 755), *F*(1,14) = 6.60, *p* = .02, η_p_^2^ = .32.

### ERP data

The grand-averaged ERPs evoked by the target event in both tasks and experimental blocks are depicted in Fig 2. Mean amplitudes and standard deviations are provided in Table 2. In both tasks, similar components were elicited by target appearance. Three time ranges were exported for further analyses: the N2 range (130 to 210 ms), the P3a range (240 to 300 ms), and the P3b range (300 to 410 ms). The same time ranges were also analyzed in our previous paper [19]. Grand averages for non-target events are depicted in Fig 3 (see Table 2 for mean amplitudes and standard deviations). Again, a similar sequence of components was elicited in both tasks. Mean amplitudes of an early N2 (100–170 ms), the P3a (240–320 ms), and P3b (320–400 ms) component were exported.

**Table 2:** Electrophysiological data of the Oddball and Cyberball group for the two experimental blocks.

		Oddball		Cyberball	
ERP component	Electrode position	Block 1	Block 2	Block 1	Block 2
**N2 (target) 130–210 ms**	**Fz**	-0.62 (1.79)	-0.31 (1.86)	0.30 (1.86)	-0.48 (3.20)
	**Cz**	-0.61 (2.18)	-0.51 (2.37)	-0.55 (2.25)	-1.78 (3.69)
	**Pz**	-1.64 (2.35)	-1.82 (2.97)	-1.62 (2.14)	-2.91 (3.45)
**P3a (target) 240–300 ms**	**Fz**	0.77 (1.89)	3.13 (2.47)	3.28 (2.86)	4.36 (5.08)
	**Cz**	3.19 (2.82)	6.59 (3.43)	4.28 (4.38)	6.93 (5.60)
	**Pz**	3.76 (2.73)	5.99 (3.71)	3.81 (4.21)	5.37 (4.26)
**P3b (target) 300–410 ms**	**Fz**	-0.77 (2.19)	1.10 (2.84)	2.13 (2.49)	2.42 (2.83)
	**Cz**	1.47 (2.51)	4.45 (3.55)	2.95 (3.70)	5.11 (2.29)
	**Pz**	3.93 (2.21)	6.99 (2.55)	3.67 (3.31)	7.05 (3.18)
**N2 (non-target) 100–170 ms**	**Fz**	0.00 (2.19)	-0.66 (1.85)	-0.07 (1.74)	-1.17 (1.53)
	**Cz**	0.58 (2.55)	-0.66 (1.97)	0.13 (1.52)	-1.30 (1.70)
	**Pz**	-0.33 (1.35)	-0.23 (1.87)	-0.26 (1.50)	-1.10 (1.49)
**P3a (non-target) 240–320 ms**	**Fz**	4.61 (4.26)	1.76 (2.39)	6.23 (2.85)	2.84 (2.40)
	**Cz**	5.15 (5.11)	1.03 (2.90)	6.62 (4.47)	2.64 (2.85)
	**Pz**	3.44 (3.48)	0.77 (2.27)	3.16 (2.88)	1.96 (2.49)
**P3b (non-target) 320–400 ms**	**Fz**	3.51 (3.93)	0.85 (2.76)	4.22 (2.01)	1.96 (2.31)
	**Cz**	3.64 (3.95)	0.34 (2.90)	3.88 (2.19)	2.09 (2.51)
	**Pz**	4.33 (1.90)	1.12 (2.49)	3.56 (2.35)	2.54 (3.29)

Mean amplitudes and standard deviations (in brackets) are presented.

#### ERP processing of target events

N2 (130–210 ms): The N2 component elicited by the presentation of the target stimulus was clearly visible in both blocks of the task, and mostly pronounced at Pz (main effect of electrode position, *F*(2,27) = 10.27, *p* < .001, η_p_² = .43). There was neither a main effect of task, *F*(1,28) = 0.13, *p* = .73, or block, *F*(1,28) = 1.20, *p* = .28, nor an interaction of the factors “block” and “task”, *F*(1,28) = 1.61, *p* = .22. Grand averages suggest an increased amplitude in the second block of the Cyberball task, but this was not statistically confirmed (analyses separate for electrode positions *p*s ≥ .11).

P3a (240–300 ms): The earlier part of the P3 complex elicited by the target stimulus was most prominent at centro-parietal leads (main effect of electrode position: *F*(2,27) = 32.56, *p* < .001, η_p_² = .71) and more pronounced in the second block (main effect of block: *F*(1,28) = 7.98, *p* = .009, η_p_² = .22). There was no main effect of task, *F*(1,28) = 0.60, *p* = .45, but the effect of block was significantly modulated by electrode position, *F*(2,27) = 7.57, *p* = .002, η_p_² = .36. Separate analyses revealed that the main effect of block was most prominent at Cz, *F*(1,28) = 10.29, *p* = .003, η_p_² = .27, as compared to Pz, *F*(1,28) = 5.25, *p* = .03, η_p_² = .16, and Fz, *F*(1,28) = 5.98, *p* = .02, η_p_² = .18. These effects were not modulated by the factor “task” at neither electrode position (*p*s ≥ .38).

P3b (300–410 ms): The P3a was followed by a P3b component, mostly pronounced at Pz (main effect of electrode position: *F*(2,27) = 31.59, *p* < .001, η_p_² = .70). A significant main effect of block indicates that the amplitude was higher in the second block of the tasks, *F*(1,28) = 21.03, *p* < .001, η_p_² = .43. There was no main effect of task, *F*(1,28) = 1.93, *p* = .18, but also the effect in the P3b range was modulated by electrode position, *F*(2,27) = 9.95, *p* = .001, η_p_² = .42. Separate analyses for the electrode positions revealed that the effect of block was most prominent at Pz, *F*(1,28) = 32.53, *p* < .001, η_p_² = .54, as compared to Cz, *F*(1,28) = 17.65, *p* < .001, η_p_² = .39, and Fz, *F*(1,28) = 4.61, *p* = .041, η_p_² = .14. These effects were not modulated by the factor “task” (*p*s ≥ .13).

#### ERP processing of non-target events

N2 (100–170 ms): The presentation of non-targets elicited an early N2 which was mostly pronounced in the second block (main effect of block: *F*(1,28) = 4.61, *p* = .04, η_p_² = .14). This effect was modulated by electrode position, *F*(2,27) = 7.98, *p* = .002, η_p_² = .37. Separate analyses revealed a main effect of block at Cz, *F*(1,28) = 7.10, *p* = .01, η_p_² = .20, but not at Fz, *F*(1,28) = 3.95, *p* = .06, or Pz, *F*(1,28) = 1.24, *p* = .27. There were neither main effects of task (*p*s ≥ .30) nor interactions with this factor (*p*s ≥ .16).

P3a (240–320 ms): The earlier part of the P3 complex elicited by the non-target stimulus was most prominent at fronto-central leads (main effect of electrode position: *F*(2,27) = 8.14, *p* = .002, η_p_² = .38), and more pronounced in the first block (main effect of block: *F*(1,28) = 34.42, *p* < .001, η_p_² = .55). There was no main effect of task, *F*(1,28) = 1.26, *p* = .27, but the effect of block was significantly modulated by electrode position, *F*(2,27) = 17.47, *p* < .001, η_p_² = .56, and by electrode position and task, *F*(2,27) = 3.47, *p* = .046, η_p_² = .21. For the Oddball task, main effects of blocks were visible at Fz, *F*(1,14) = 13.63, *p* = .002, η_p_² = .49, Cz, *F*(1,14) = 16.52, *p* = .001, η_p_² = .54, and Pz, *F*(1,14) = 12.11, *p* = .004, η_p_² = .46. However, the effect of block was restricted to Fz, *F*(1,14) = 32.01, *p* < .001, η_p_² = .70, and Cz, *F*(1,14) = 21.66, *p* < .001, η_p_² = .61, in the Cyberball task (Pz: *F*(1,14) = 3.35, *p* = .089, η_p_² = .19).

P3b (320–400 ms): The P3a was followed by a P3b component which was significantly reduced in the second block, *F*(1,28) = 26.97, *p* < .001, η_p_² = .49. This effect was not modulated by task, *F*(1,28) = 2.22, *p* = .15, or by task and electrode position, *F*(2,27) = 1.65, *p* = .21. In addition, there was no main effect of task, *F*(1,28) = 0.88, *p* = .36.

## Discussion

### Summary of results

In line with the hypotheses, the reduction of target probability did not lead to increased feelings of exclusion or negative mood in the Oddball task as it did in the Cyberball task. At the same time, participants of both groups were able to notice the difference of target probabilities between the two blocks. The target event triggered an N2/P3 complex in both paradigms. Reduced target probability did not affect the N2 amplitude, but increased P3a and P3b amplitudes in both the Oddball and Cyberball task. The most prominent component triggered by the non-target events was a fronto-central P3a, followed by a smaller P3b component. Both components were affected by stimulus probability in both tasks: they were less pronounced in the second compared to the first block. An early N2 was enhanced in the second block. This effect was not modulated by the factor “task”. Results show that the ERP effects are mainly independent from social context or perceived social exclusion.

### Questionnaire data

In Cyberball studies, social exclusion is defined by reducing the probability of ball receptions which led to perceived ostracism in total (e.g. [7, 16]) and partial (e.g. [3, 18]) exclusion paradigms. The data of the Cyberball group replicated these results: after exclusion, participants reported a decrease in need satisfaction and an increase in negative mood. However, the reduction of the scale “meaningful existence” did not reach significance. Although there are reports that not all needs are similarly affected in paradigms using computer-mediated communication [32], we suggest that this missing effect is due to the small sample size at least for questionnaire data.

On the contrary, the results of the visual Oddball task show that by eliminating the social context reduced target probability–which was clearly noticed by the participants–did not lead to a feeling of social exclusion. Although there is evidence that the credibility of the cover story or the source of exclusion have minimal influence on the induction of social need threat during Cyberball [19, 31], the complete removal of the social context eliminated the robust exclusion effect.

The reduction of target probabilities was noticed in both groups. However, the Cyberball group was more sensitive with respect to the perceived difference in target probability. This task effect could be due to discrepancies in the instructions and experimental procedure: in the Cyberball task, participants were asked to imagine themselves in two different situations of a ball-tossing game (in the meadow and on a beach), so that it would be easier for them to differentiate between blocks while retrospectively filling out the questionnaires after the second block of the game. In contrast, participants in the Oddball group were told that they took part in a simple reaction task, and an additional imaging task was not required. This might have diminished the differentiation between blocks. However, this modulation by the factor “task” did not lead to a different processing of the target events on the level of ERPs which will be discussed in detail in the following.

### ERP data: targets

A direct comparison of ERP results obtained in the Cyberball and Oddball task showed that the processing of target events (“ball possession” in Cyberball) did not differ: two components were elicited by the presentation of this event–a parietal N2, and a P3 complex constituted by a centro-parietal P3a and a parietal P3b.

Since the N2 component was not affected by the reduction of target probability, we suggest that this component reflects task relevance or target detection: it was shown previously that targets compared to non-targets and ball receptions compared to non-ball receptions elicited a posterior N2 [18, 27, 33]. This component is not sensitive to the probability of a task-relevant event–the slight enhancement in the Cyberball group did not reach significance which replicates previous findings [18, 23]. The similarity of the N2 components elicited in both the Cyberball and Oddball group also indicates that the relevance of the target stimulus does not differ between paradigms.

The effects of target probability on the P3 complex in the visual oddball task were similar to the effects obtained during Cyberball. The P3 complex in our previous study was divided into a more central P3a and a parietal P3b [18] which were both affected by the probability of ball reception. In that study the P3a effect was found to be correlated with an increase in negative mood and the P3b effect was correlated with an increase in perceived ostracism intensity [18]. Although the relationship between the P3 components and stimulus probability was confirmed in our current study, the ERP effects were not modulated by the factor “task”: the increase in P3 amplitude driven by a reduction in target probability was also obtained if need satisfaction or negative mood remained constant–in the Oddball task. Moreover, the ERP effects were not consistently correlated with the expression of the NTQ effect–in the Cyberball task (see S1 Table for *r* and *p* values for both experimental groups). Both observations question the relation of the ERP effects to need threat, since the effects in the P3 range are not necessarily associated with the experience of social exclusion. The P3 effect rather corresponds to the estimation of target probability and the subjective expectancy for target appearance [34, 35], which can therefore serve as the most parsimonious explanation within the Cyberball paradigm. This is also in line with a previous study in which P3 amplitudes elicited by the event “self” during “overinclusion” (46% ball possession) were reduced as compared to inclusion [36].

### ERP data: non-targets

Non-target events elicited two distinct components in the Cyberball and Oddball task which were affected by the factor “block”: an early N2 component and a P3 complex with a fronto-central P3a and a following–more widespread–P3b.

The N2 elicited by non-targets was more pronounced in the second block of both tasks. This effect was restricted to the N2 recorded at Cz, but it was not modulated by the factor “task”. It is probably related to differentially pronounced response preparation processes between blocks [37], but this should be clarified in future studies. An earlier Cyberball study found a frontal N2 which was elicited by “other” trials in both exclusion and inclusion blocks. It was related to a conflict-based neural alarm activation [16]. Since the N2 response to non-targets was elicited in our study in the Cyberball and the Oddball task, the underlying process is unlikely to reflect the activation of an alarm system. We propose that the fronto-central N2 is rather linked to inhibition processes, previously reported in go/no-go paradigms [37].

The P3 complex elicited by non-targets had a more anterior topography as compared to the target P3s. This is in line with a recent Cyberball study [22] where an anterior P3a was more pronounced for “exclusionary” (= non-target) trials during inclusion as compared to “inclusionary” (= target) trials. Grand averages in our current study suggest that the P3a is more pronounced for “exclusionary” trials and the P3b is more pronounced for “inclusionary” trials. These effects are well-known in oddball paradigms [28]. In addition, the reduced P3a amplitudes in the second block (66% non-target probability) as compared to the first block (33% non-target probability) replicate the findings of the recent Cyberball study mentioned above [22]: in this study the P3a decreased during continuous exclusion which followed an inclusion block. We suggest that this effect is due to the increased probability of “other” events during exclusion and a corresponding adaptation of expectancies for these events. However, we have to consider that the effects in the P3a range slightly differ between tasks: Despite the restricted number of electrodes, the topography of the effect of block on P3a amplitudes in the Cyberball task appears to be more shifted towards frontal leads. Whether this shift indicates the involvement of different processes remains to be answered (see also “Limitations and future directions”).

In a previous Cyberball study the P3b triggered by the event “other” was more pronounced during inclusion as compared to exclusion [17]. According to Kawamoto and colleagues, the enhanced P3b amplitude was related to a modulation of the attentional focus on exclusionary cues which–in turn–led to greater social pain. Our data suggest that the P3b amplitude triggered by these events–comparable to the “self” events–is a function of stimulus probability: the event “ball possession of the co-player” is more frequent in the exclusion condition compared to the inclusion condition. A direct comparison with the Oddball task in the current study underlines this assumption. Similar results were also obtained in independent oddball studies for non-target events [26, 38].

In the Cyberball studies mentioned above the P3 effect was correlated with an increase in social pain [17], or an decrease in positive affect and control [16]. We also calculated correlations between ERP components affected by the factor “block” and questionnaire data separately for both paradigms, but we did not find reliable effects (see S1 Table and also [21, 23]). This supports the results of the direct comparison of Cyberball and Oddball: P3 effects can be dissociated from a feeling of exclusion. Our data indicate that ERP effects obtained in the Cyberball paradigm are not necessarily related to the activation of an exclusion-specific neural alarm system or to the level of need threat [1, 18] since they can also be elicited by a physically similar visual oddball task lacking any social cues. They rather seem to reflect basal cognitive functions and thereby are in line with alternative explanations for the neural effects of exclusion obtained in fMRI studies [12, 13]. We suggest that expectancy-related cognitive processes account for the effects (see also [23]): during exclusion not receiving the ball meets one’s expectation of exclusion, and occasionally receiving it contradicts them which are both reflected in the P3 amplitudes of “self” and “other” events. Additional evidence is given by a recent Cyberball study with a clinical sample: the more the current expectancy for involvement was violated by receiving the ball, the more the P3 amplitude was pronounced [39]. It was also suggested by a model of social exclusion that “exclusion detectors” are only triggered in situations where the behavior of an interaction partner is more exclusionary than expected [40]. However, our data show that ERP effects in the N2 and P3 range are mainly independent from social context or perceived social exclusion. This fits well into a recently proposed theory which states that both low level (e.g. in Stroop tasks) and high level (e.g. cognitive dissonance) inconsistencies, i.e. deviations from current expectancies, are detected by a common neuronal network [41].

### Limitations and future directions

Despite the striking similarities between the ERP effects obtained in the Cyberball and Oddball task–especially for the processing of target events–we have to consider that the topography of effects on P3a amplitudes elicited by non-targets slightly differs between tasks. This shift in topography towards frontal leads in the Cyberball task has to be confirmed by multi-electrode recordings to explore if different neural networks are involved. Although this shift is the only significant difference of ERP effects between tasks, grand averages–with respect to the P3b –suggest that the effect of probability was more pronounced in the Oddball task as compared to the Cyberball task (see Fig 3). Even though not significant, this trend contrasts our previous results where we found a distinct effect of probability on parietal P3b amplitudes elicited by “other” trials during Cyberball [23]. This modulation could be due to methodological differences: in our previous study there were 300 ball throws, in our current study there were only 200 trials per block. It is possible that the adjustment of expectancies takes longer in a Cyberball game as compared to an Oddball task which would then lead to a delayed reduction of P3b amplitudes in Cyberball as compared to Oddball.

In a previous ERP Cyberball study late (> 400 ms after stimulus appearance) frontal components were related to perceived distress [20]. We have analyzed the descriptively visible difference at around 450 ms for the target events, but we did not find an effect of task or a correlation between this component and perceived need threat. This could be due to the small sample size or–more likely–due to the rather short time-lag between relevant events in our study which probably led to a superimposition of slow waves by the ERPs elicited by the following event. Therefore a valid analysis of late–and slow–ERP components is questionable in our design (see also [22]). A possible effect on a late frontal negativity in our study could be related to conflict processing [42]. We tentatively suggest that conflict during response selection could have been higher in the second block of the Cyberball task: it could be possible that the participants tried to figure out a strategy how to play to influence the behavior of their co-players to get included again. This would also explain the prolongation of reaction times in the second block of the Cyberball task which was not observed in the Oddball task. Further studies will have to show whether late ERP components can be validly and reliably related to the affective evaluation of the expectancy violation signaled by the P3 complex.

Another important point is if our findings are specifically related to the Cyberball paradigm. Although we conclude that expectancy violations account for the effects obtained in this paradigm–on the level of probability sensitive ERP components and also on the level of questionnaire data [23]–we cannot rule out that social pain can be elicited in other paradigms by mechanisms apart from expectancy violations. Unfortunately, the definition of “social pain” is rather broad, referring to the “unpleasant experience that is associated with actual or potential damage to one’s sense of social connection or social value (owing to social rejection, exclusion, negative social evaluation or loss)” ([10], p. 421). Eisenberger’s definition shows that ostracism is a complex social phenomenon, but nevertheless some of the underlying mechanism can rely on basal cognitive functioning. Efforts should be made to distinctively investigate these mechanisms to identify emotional and cognitive processes which are either specifically “social” or part of a more general network.

## Supporting Information

S1 Table Correlations between the experimental effects (Δ block 1, block 2) of questionnaire and ERP data. (DOCX)
