# Supplementary material for: Target and Non-Target Processing during Oddball and Cyberball: A Comparative Event-Related Potential Study
Source: PLoS One. 2016 Apr 21;11(4):e0153941. doi: 10.1371/journal.pone.0153941 (PMC4839683; doi:10.1371/journal.pone.0153941)
Supplement: S1 Table — (DOCX) [file pone.0153941.s001.docx]

**S1 Table. Correlations between the experimental effects (Δ block 1, block 2) of questionnaire and ERP data.**

|  | | **Oddball** | | | **Cyberball** | | |
| --- | --- | --- | --- | --- | --- | --- | --- |
| **ERP component** | **NTQ scale** | **Fz** | **Cz** | **Pz** | **Fz** | **Cz** | **Pz** |
| **N2 (target)**  **130-210 ms** | Estimated target occ. | -.384 (.158) | -.172 (.540) | .304 (.270) | -.487 (.066) | -.529* (.043) | -.357 (.191) |
|  | Belonging | -.352 (.199) | -.279 (.314) | -.271 (.329) | -.247 (.375) | -.355 (.195) | -.258 (.353) |
|  | Self-esteem | .007 (.981) | .227 (.415) | .300 (.278) | -.052 (.855) | .078 (.783) | .331 (.228) |
|  | Meaningful existence | -.023 (.937) | .146 (.604) | .383 (.158) | -.551* (.033) | -.519* (.047) | -.294 (.288) |
|  | Control | .250 (.369) | .349 (.202) | .044 (.877) | -.144 (.609) | -.146 (.605) | .023 (.934) |
|  | Negative mood | .302 (.273) | .363 (.183) | .352 (.198) | -.302 (.274) | -.332 (.227) | -.135 (.630) |
| **P3a (target)**  **240-300 ms** | Estimated target occ. | -.092 (.743) | -.119 (.674) | -.104 (.711) | -.348 (.203) | -.412 (.127) | -.284 (.305) |
|  | Belonging | -.060 (.832) | -.282 (.309) | -.481 (.069) | -.140 (.619) | -.173 (.538) | .075 (.791) |
|  | Self-esteem | .087 (.757) | .062 (.827) | .064 (.821) | .123 (.663) | .163 (.562) | .035 (.902) |
|  | Meaningful existence | -.383 (.158) | -.335 (.222) | -.226 (.419) | -.250 (.368) | -.271 (.328) | -.284 (.304) |
|  | Control | .117 (.679) | .078 (.782) | -.096 (.732) | .041 (.886) | .008 (.978) | .127 (.653) |
|  | Negative mood | .060 (.832) | -.067 (.811) | -.246 (.377) | -.032 (.909) | -.089 (.752) | -.002 (.995) |
| **P3b (target)**  **300-410 ms** | Estimated target occ. | .079 (.780) | -.004 (.988) | -.047 (.869) | -.657** (.008) | -.416 (.123) | .159 (.573) |
|  | Belonging | .011 (.968) | -.096 (.733) | -.199 (.477) | -.420 (.119) | -.258 (.353) | .323 (.240) |
|  | Self-esteem | -.030 (.915) | -.019 (.947) | -.102 (.716) | -.200 (.476) | -.116 (.681) | .048 (.866) |
|  | Meaningful existence | -.210 (.452) | -.331 (.228) | -.344 (.210) | -.250 (.369) | -.040 (.886) | .223 (.425) |
|  | Control | .013 (.963) | .141 (.617) | .007 (.981) | -.287 (.299) | -.309 (.262) | .151 (.591) |
|  | Negative mood | .004 (.989) | .076 (.788) | -.057 (.841) | -.148 (.598) | -.002 (.996) | .355 (.194) |
| **N2 (non-target)**  **100-170 ms** | Estimated target occ. | .401 (.139) | .331 (.228) | .101 (.720) | -.066 (.816) | -.092 (.746) | -.121 (.667) |
|  | Belonging | -.089 (.753) | -.018 (.950) | .200 (.476) | .105 (.709) | -.027 (.925) | -.114 (.686) |
|  | Self-esteem | -.127 (.651) | -.156 (.578) | -.290 (.294) | -.449 (.093) | -.259 (.352) | -.179 (.524) |
|  | Meaningful existence | -.148 (.599) | -.283 (.306) | -.409 (.130) | -.311 (.259) | -.191 (.495) | -.241 (.388) |
|  | Control | .345 (.208) | .295 (.286) | .350 (.200) | -.125 (.656) | .061 (.829) | .083 (.770) |
|  | Negative mood | .214 (.444) | .284 (.305) | .213 (.446) | .088 (.756) | .049 (.861) | -.128 (.649) |
| **P3a (non-target)**  **240-320 ms** | Estimated target occ. | .236 (.397) | .455 (.088) | .404 (.135) | -.164 (.560) | .039 (.891) | -.009 (.973) |
|  | Belonging | -.071 (.801) | -.125 (.656) | -.144 (.610) | .258 (.353) | .225 (.420) | .240 (.388) |
|  | Self-esteem | -.167 (.551) | -.118 (.675) | -.102 (.718) | -.219 (.433) | -.184 (.512) | -.529* (.043) |
|  | Meaningful existence | .004 (.989) | -.090 (.750) | -.279 (.314) | .215 (.441) | .149 (.597) | -.045 (.874) |
|  | Control | .110 (.697) | .146 (.604) | .115 (.683) | .313 (.256) | .378 (.164) | .192 (.492) |
|  | Negative mood | -.180 (.522) | -.059 (.835) | -.287 (.299) | .232 (.406) | .124 (.661) | -.070 (.805) |
| **P3b (non-target)**  **320-400 ms** | Estimated target occ. | .092 (.743) | .211 (.451) | .137 (.626) | -.055 (.847) | .115 (.683) | .196 (.483) |
|  | Belonging | .053 (.852) | .056 (.843) | .146 (.604) | .371 (.173) | .405 (.134) | .340 (.215) |
|  | Self-esteem | -.344 (.209) | -.379 (.164) | -.310 (.262) | -.352 (.198) | -.356 (.192) | -.359 (.189) |
|  | Meaningful existence | .048 (.865) | -.156 (.579) | -.278 (.316) | .022 (.938) | -.089 (.753) | -.077 (.786) |
|  | Control | .098 (.728) | .032 (.910) | -.277 (.318) | .157 (.576) | .220 (.430) | .139 (.621) |
|  | Negative mood | .008 (.978) | -.229 (.412) | -.494 (.062) | .266 (.338) | .185 (.510) | .190 (.497) |

**Note.** Correlations between the experimental effects (Δ block 1, block 2) of questionnaire (NTQ scales) and ERP data for the Oddball and Cyberball group and the three electrode positions are presented. For “estimated target occurrence”, “belonging”, “self-esteem”, “meaningful existence”, and “control” the differences is “block 1 – block 2”. For “negative mood” and for all ERP data the difference is “block 2 – block 1”. Bivariate (Pearson) correlations (*r*) and *p* values (in brackets) are presented. * = *p* < .05, ** = *p* < .01.
